# *Fusobacterium nucleatum* drives a pro-inflammatory intestinal microenvironment through metabolite receptor-dependent modulation of IL-17 expression

**DOI:** 10.1080/19490976.2021.1987780

**Published:** 2021-11-15

**Authors:** Caitlin A. Brennan, Slater L. Clay, Sydney L. Lavoie, Sena Bae, Jessica K. Lang, Diogo Fonseca-Pereira, Kathryn G. Rosinski, Nora Ou, Jonathan N. Glickman, Wendy S. Garrett

**Affiliations:** a Department of Immunology and Infectious Diseases, Harvard T. H. Chan School of Public Health, Boston, Massachusetts; b Harvard T. H. Chan Microbiome in Public Health Center, Boston, Massachusetts; c Department of Pathology, Harvard Medical School, Boston, Massachusetts; d Beth Israel Deaconess Medical Center, Boston, Massachusetts; e Broad Institute of Harvard and MIT, Cambridge, Massachusetts; f Department and Division of Medical Oncology, Dana-Farber Cancer Institute and Harvard Medical School, Boston, Massachusetts; g Department of Molecular Metabolism, Harvard T. H. Chan School of Public Health, Boston, Massachusetts

**Keywords:** Microbiome, colorectal cancer, Th17 cells, interleukin 17, gnotobiotics, Fusobacterium nucleatum, altered Schaedler’s flora

## Abstract

The colorectal cancer (CRC)-associated microbiota creates a pro-tumorigenic intestinal milieu and shapes immune responses within the tumor microenvironment. However, how oncomicrobes – like *Fusobacterium nucleatum*, found in the oral cavity and associated with CRC tissues*–* affect these distinct aspects of tumorigenesis is difficult to parse. Herein, we found that neonatal inoculation of *Apc^Min/+^* mice with *F. nucleatum* strain Fn7-1 circumvents technical barriers preventing its intestinal colonization, drives colonic *Il17a* expression prior to tumor formation, and potentiates intestinal tumorigenesis. Using gnotobiotic mice colonized with a minimal complexity microbiota (the altered Schaedler’s flora), we observed that intestinal Fn7-1 colonization increases colonic Th17 cell frequency and their IL-17A and IL-17F expression, along with a concurrent increase in colonic lamina propria *Il23p19* expression. As Fn7-1 stably colonizes the intestinal tract in our models, we posited that microbial metabolites, specifically short-chain fatty acids (SCFA) that *F. nucleatum* abundantly produces in culture and, as we demonstrate, in the intestinal tract, might mediate part of its immunomodulatory effects *in vivo*. Supporting this hypothesis, we found that Fn7-1 did not alter RORγt^+^ CD4^+^T cell frequency in the absence of the SCFA receptor FFAR2. Taken together, our work suggests that *F. nucleatum* influences intestinal immunity by shaping Th17 responses in an FFAR2-dependent manner, although further studies are necessary to clarify the precise and multifaceted roles of FFAR2. The potential to increase intestinal Th17 responses is shared by another oncomicrobe, enterotoxigenic *Bacteroides fragilis*, highlighting a conserved pathway that could potentially be targeted to slow oncomicrobe-mediated CRC.

## Introduction

Colorectal cancer (CRC), the third leading cause of cancer-related deaths, is driven by host genetics and environmental factors. Specific bacteria, such as *Fusobacterium nucleatum*, enterotoxigenic *Bacteroides fragilis* (ETBF), and colibactin-producing *Escherichia coli*, are associated with human CRC and influence tumor multiplicity in pre-clinical murine models.^1–3^ How these bacteria potentiate tumorigenesis varies among these microbes: colibactin-producing *E. coli* expresses a genotoxin that induces a specific mutational signature found within genomes from human CRC tissues,^4,5^ and ETBF and *F. nucleatum* shape pro-inflammatory and pro-tumorigenic immune environments in the intestinal tract or within the tumor microenvironment.^1,2,6,7^ Diverse immunological responses – including production of cytokines like TNFα, IL-6, IL-1β, IL-23, and IL-17, all of which can be triggered by microbes or their products – contribute to the progression of intestinal tumorigenesis.^8^ Unlike ETBF, however, *F. nucleatum* encodes no obvious toxin that might influence such immune responses. Fusobacterial adhesins such as Fap2, FadA, and CbpF, which engage human TIGIT to mediate anti-tumor immune evasion, E-cadherin to activate β-catenin signaling, and CEACAM1 to alter T cell function, respectively, have been implicated as mediators of its tumor-permissive effects.^9–11^ Further, *F. nucleatum* lipopolysaccharide, the microbe-associated molecular pattern produced by most Gram-negative bacteria, triggers Toll-like receptor signaling, leading to NF−κB activation and microRNA21 expression, which can contribute to colitis-associated cancer development.^12,13^ However, what other fusobacterial factors, such as immunomodulatory metabolites, might contribute to *F. nucleatum*’s role in CRC development remain under-explored.

*F. nucleatum* is a normal constituent of the human oral cavity, where it influences biofilms and pro-inflammatory cytokine production that contribute to periodontitis and gingivitis.^14^ *F. nucleatum* drives a similar pro-inflammatory cytokine signature in culture using cancer cell lines and in murine preclinical models of intestinal tumorigenesis and inflammation. This signature includes upregulation of IL-8 (encoded by *Scyb1* in mice), IL-6, TNF, and COX-2 (encoded by *Ptgs2*) among others, after exposure to *F. nucleatum*.^1,7,10,15^ However, these models often involve short exposure to *F. nucleatum* (as in cell culture experiments), repeated inoculation, or antibiotic treatment, suggesting that *F. nucleatum* is unable to maintain a stable niche in the homeostatic murine intestinal tract and consistent with human studies in which *F. nucleatum* is rarely a member of the healthy stool microbiota. Under these conditions, *F. nucleatum* is unlikely to be actively growing and therefore may not producing metabolites that could tune the pre-tumoral immune environment.

We sought to understand how *F. nucleatum* and its metabolites, in particular its high production of short-chain fatty acids (SCFA) like acetate and butyrate, contribute to the development of a pro-inflammatory, pro-tumorigenic intestinal environment. To mitigate the limitations of current models, we leveraged neonatal inoculation of specific-pathogen free mice as well as gnotobiotics to develop preclinical models allowing *F. nucleatum* to gain a foothold in an otherwise restrictive intestinal niche, with the overarching goal to reveal how *F. nucleatum* influences the intestinal immune environment prior to tumor development. These models enabled us to uncover a role for *F. nucleatum* in shaping Th17 responses in the intestinal tract, providing a target for future preventative and therapeutic interventions for *F. nucleatum*-associated CRC.

## Results

### *Neonatal inoculation of* Apc^Min/+^ *mice with* F. nucleatum *Fn7-1 increases colonic tumor multiplicity and drives* Il17a *expression prior to tumor formation*

**Figure 1.**
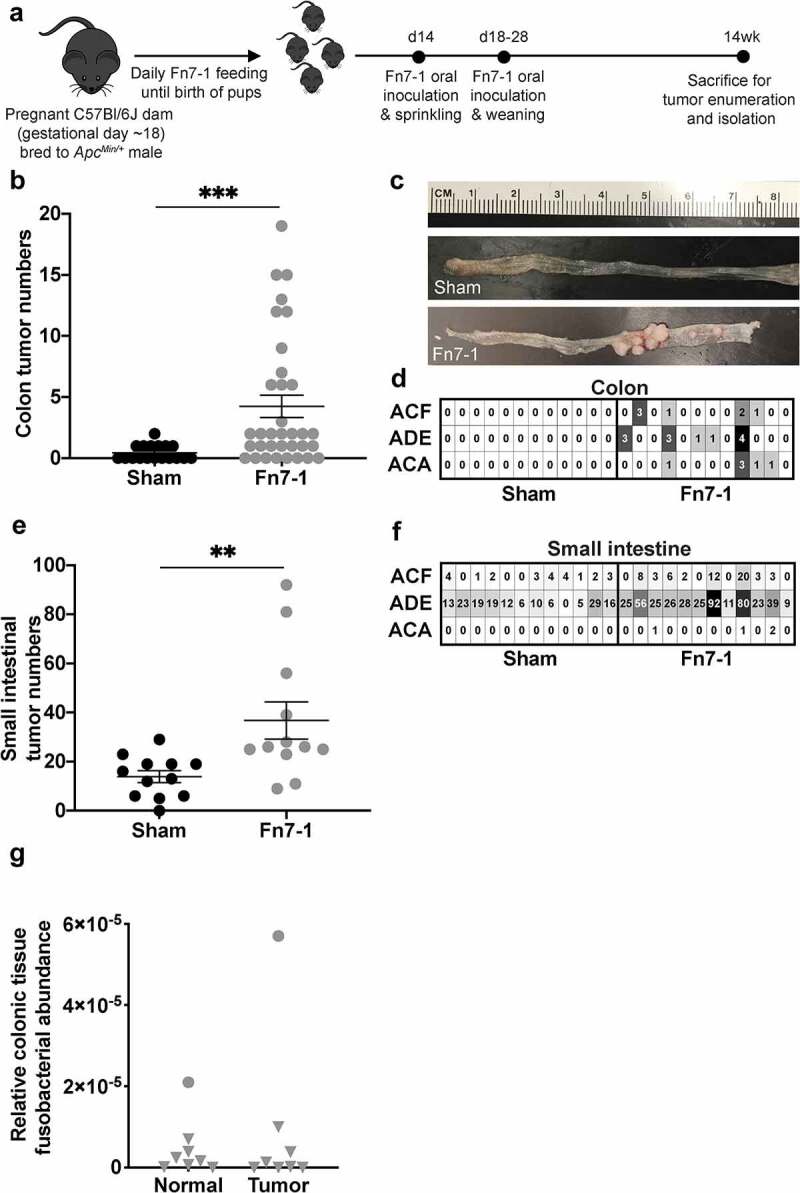
**Neonatal exposure of SPF *Apc^Min/+^* mice with Fn7-1 promotes intestinal tumor formation**. (a) Neonatal exposure model schematic. (b) Colonic tumor burdens from sham or Fn7-1 neonatally exposed *Apc^Min/+^* mice at 14 weeks. (c) Representative photographs of the colons of sham or Fn7-1 neonatally exposed *Apc^Min/+^* mice. (d) Blinded assessment of H&E-stained colonic tissues from sham or Fn7-1 neonatally exposed *Apc^Min/+^* mice to identify aberrant crypt foci (ACF), adenomas (ADE), and adenocarcinomas (ACA). Each column represents an individual mouse. (e) Small intestinal tumor burdens from sham or Fn7-1 neonatally exposed *Apc^Min/+^* mice at 14 weeks. (f) Blinded assessment of H&E-stained small intestinal tissues from sham or Fn7-1 neonatally exposed *Apc^Min/+^* mice to identify ACF, ADE, and ACA. Each column represents an individual mouse. (g) Fusobacterial abundance, determined by probe-based qPCR on DNA from colonic normal and tumor tissue of Fn7-1 neonatally exposed *Apc^Min/+^* mice and normalized to mouse *ActB* copies, using primers listed in Supplemental Table S1. Data presented are calculated as 2^^^-(CT*_F. nucleatum nusG_ –* CT*_mActB_*). Samples with no detectable *Fusobacterium*, as determined by the *F. nucleatum nusG* cycle threshold (CT) compared to a control without *F. nucleatum*, are indicated by a triangle. All data points reflect data from an individual mouse or sample, with error bars indicating mean ± standard error of the mean (SEM), and statistics were calculated using a Mann-Whitney test. ** indicates *P* < .01; *** indicates *P* < .001

We began by establishing a system of *F. nucleatum* colonization to determine how metabolically active Fn7-1 might affect intestinal tumor progression. We orally instilled pregnant dams from our conventionally-reared, specific-pathogen-free (SPF) *Apc^Min/+^* breeding colony with Fn7-1 (an intestinal isolate from a patient with Crohn’s disease that potentiates intestinal tumorigenesis in other models^1,16^), or a medium control (sham) in the days leading up to birth, beginning at gestational day ~18 (Figure 1a). We then orally inoculated and sprinkled the pups with Fn7-1 at post-natal day 14, and again orally inoculated at weaning. We hypothesized that this inoculation schema would allow Fn7-1 to gain a niche in the intestinal tract of these mice before they developed a more complex microbiota and immune system. This approach is in contrast to earlier studies wherein *Apc^Min/+^* mice were orally inoculated daily with Fn7-1 for several months.^1^ We then allowed these neonatally inoculated mice to age until 14 weeks of age and assessed their intestinal tumor burden (Figure 1(b–f)). Mice neonatally inoculated with Fn7-1 developed significantly more colonic and small intestinal tumors than sham-treated mice. There were also more neoplastically advanced lesions in Fn7-1 neonatally inoculated mice, specifically intramucosal adenocarcinomas, which we rarely detect in untreated *Apc^Min/+^* mice. These results demonstrate that Fn7-1 neonatal inoculation of *Apc^Min/+^* mice affects not only intestinal tumor burden but also neoplastic progression.

However, in contrast with daily-fed *Apc^Min/+^* mice,^1^ colonic tumor and normal tissues from neonatally inoculated mice harbored minimal, if any, *F. nucleatum* (Figure 1g). *F. nucleatum* burden was determined using probe-based quantitative PCR (qPCR) on DNA from colonic normal and tumor tissue of Fn7-1 neonatally exposed *Apc^Min/+^* mice, using previously characterized primers that target *F. nucleatum nusG*^17,18^ and mouse *ActB*, to normalize to tissue DNA copies. This result suggests that neonatal inoculation serves as a model for Fn7-1’s pro-tumorigenic effect without confounding intratumoral Fn7-1’s shaping of the established tumor microenvironment, allowing us to delineate these two important steps in how *F. nucleatum* may influence CRC progression.

**Figure 2.**
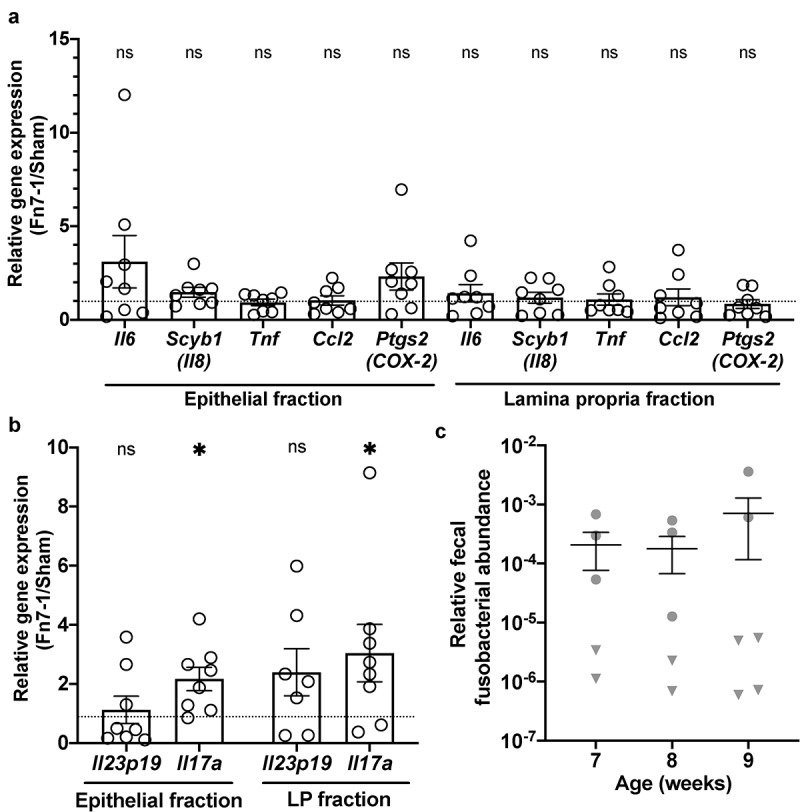
**Neonatal inoculation with Fn7-1 leads to increased *Il17a* expression in the colonic lamina propria prior to tumor formation but does not alter gene expression of the *F. nucleatum*-associated pro-inflammatory signature**. (a) Relative expression of *Il6, Scyb1* (*Il8*), *Tnf, Ccl2* and *Ptgs2* (*COX-2*), as determined by RT-qPCR, from colonic epithelial and LP fractions of Fn7-1 or sham neonatally inoculated *Apc^Min/+^* mice at 7–9 weeks of age. (b) Relative expression of *Il23p19* and *Il17a*, as determined by RT-qPCR, in colonic epithelial and LP fractions of Fn7-1 or sham neonatally inoculated *Apc^Min/+^* mice. (c) Relative fusobacterial abundance, normalized to total 16S, as determined by SYBR qPCR on DNA isolated from the feces of Fn7-1-neonatally inoculated mice at the indicated time points. Data presented are calculated as 2^^^-(CT*_Fusobacterium_*_16S_ – CT_eubacterial 16S_). Fecal samples with no detectable *Fusobacterium*, as determined by the *Fusobacterium* 16S CT compared to a control without *F. nucleatum*, are indicated by a triangle. All data points reflect the data from an individual mouse or sample, with error bars indicating mean ± SEM, and statistics were calculated using a Wilcoxon signed-rank test compared to a null hypothesis of 1. * indicates *P* < .05; ns indicates not significant

To understand how Fn7-1 might promote tumor formation, we hypothesized that Fn7-1 exerts pro-inflammatory effects in the colon prior to tumor formation as has been demonstrated for ETBF.^2^ As another study has demonstrated that the microbiota distinctly contributes to colonic but not small intestinal tumorigenesis,^19^ herein we focused on how Fn7-1 alters the colon immune environment. We examined gene expression in the colonic lamina propria (LP) and epithelial fractions of mice neonatally colonized with Fn7-1 or sham at 7–9 weeks of age, prior to the development of macroscopic tumors. Focusing on the pro-inflammatory genes that have been associated with *F. nucleatum*, we observed minimal effects on expression of *Il6, Scyb1 (Il8), Tnf, Ccl2*, or *Ptgs2* (*COX-2*) in either the colonic epithelial or LP fractions in Fn7-1 exposed mice (Figure 2a). Although these genes are often upregulated in response to *F. nucleatum* exposure, these results led us to reconsider the pro-inflammatory pathways Fn7-1 might influence to contribute to the development of a pro-tumorigenic environment in this model. ETBF influences intestinal tumorigenesis through the IL-23/IL-17 axis.^2,6^ IL-23 is a pro-inflammatory cytokine that drives the expansion of IL-17-expressing cells such as T helper 17 (Th17) in the intestinal tract. To ascertain whether Fn7-1 might exert similar effects, we assessed *Il23p19* and *Il17a* expression in the colonic epithelium and LP. While we did not see a statistically significant increase in *Il23p19* expression, we observed upregulation of *Il17a* in both the colonic epithelium and LP of mice neonatally inoculated with Fn7-1 (Figure 2b). We also confirmed that *F. nucleatum* could still be detected in the feces of mice at this time point (Figure 2c), as determined by qPCR performed on fecal DNA using primers targeting *Fusobacterium spp*. 16S rDNA^1,20^ and normalized to total eubacterial 16S rDNA copies. However, we only detected *F. nucleatum* in a subset of these mice, which may explain the variability we observed in this model. These results suggest that, prior to tumor development and when Fn7-1 is still colonizing the intestinal tract, Fn7-1 modulates the intestinal immune environment by upregulating *Il17a* expression. However, it was not clear what IL-17-expressing cell population(s) drive this change.

### F. nucleatum *colonizes gnotobiotic altered Schaedler’s flora mice without inducing colitis or altering ASF community member abundance*

Our observations in the neonatal inoculation model led us to explore how Fn7-1 might induce IL-17 in the intestinal tract. However, experimental limitations of the neonatal inoculation model, including the variability of Fn7-1 levels across mice (Figure 2c), drove us to use gnotobiotic mice, which are specially reared to control the micro-organisms to which they are exposed and harbor. Specifically, we used mice colonized with the altered Schaedler’s flora (ASF). The ASF are a community of eight murine-isolated bacterial strains that are stably maintained in the mouse gut, allowing transgenerational community reproducibility and providing microbial cues for more normal physiology and immune system development than germ-free mice.^21–23^ The ASF’s minimal microbial complexity also allows investigators to circumvent the technical limitation some bacteria, like *F. nucleatum*, engender as they are unable to colonize mice with a more complex microbiota and therefore are difficult to study in conventionally reared SPF mice. We also utilized wild-type C57B/6J mice, rather than *Apc^Min^*^/+^, to enable us to study how Fn7-1 shapes the colonic immune responses without the confounding effects of this genetic mutation and any precancerous lesions that could influence intestinal immune responses.

**Figure 3.**
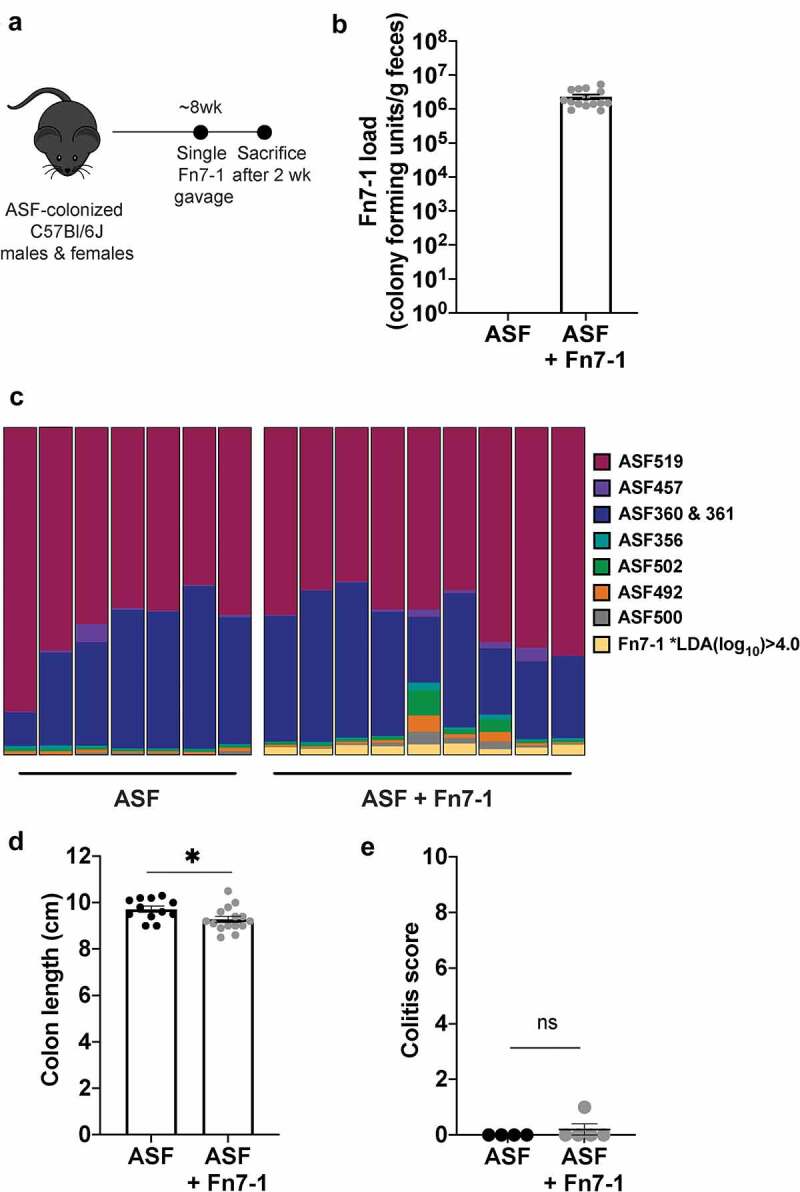
**Fn7-1 colonizes ASF mice without altering community structure or inducing colitis**. (a) ASF+Fn7-1 experimental schematic. (b) Colony-forming units of Fn7-1 per g feces after 2 weeks of colonization of ASF mice. (c) Fecal community structure from ASF and ASF+Fn7-1 mice as determined by 16S rRNA amplicon sequencing and analyzed by QIIME2 with DADA2. Each column reflects the proportion of different community members from an individual mouse (additional data in Supplemental Table S2). Only *F. nucleatum* abundance significantly differs across the two groups (LEfSe analysis, LDA(log_10_) > 4.0). (ASF519: *Parabacteroides sp*.; ASF457: *Mucispirillum sp*.; ASF360 and ASF361: *Lactobacillus spp*.; ASF356 and ASF502: *Lachnospiraceae* family members; ASF492: *Eubacterium sp*.; and ASF500: related to *Colidextribacter sp*.) (d) Colon length from ASF and ASF+Fn7-1 mice. (e) Histological colitis analysis of colons from ASF and ASF+Fn7-1 mice. All data points reflect the data from an individual mouse or sample, with error bars indicating mean ± SEM, and statistics were calculated using a Mann-Whitney test. * indicates *P* < .05; ns indicates not significant

In this approach, a single gavage of Fn7-1 was sufficient to colonize ASF mice to consistent and high levels by 2 weeks post-inoculation, even enabling detection of fecal *F. nucleatum* by the gold standard of bacterial enumeration – colony-forming units (Figure 3(a,b)). We next performed 16S rRNA gene amplicon sequencing on DNA isolated from the stool of ASF mice and ASF mice gavaged with Fn7-1 (referred to herein as ASF+Fn7-1 mice). We detected reproducible levels of *F. nucleatum* in ASF+Fn7-1 mice (~2–3% of the reads; Figure 3c & Supplemental Table S2). To ascertain if Fn7-1 colonization shapes the abundance of the other ASF members, we performed LEfSe linear discriminant analysis^24^ and found *F. nucleatum* to be the only OTU to significantly differ between ASF and ASF+Fn7-1 mice. We also dissected the colons for measurement and histological analysis to determine whether Fn7-1 colonization leads to intestinal inflammation in these mice. While we observed a subtle, but statistically significant, decrease in colon length after Fn7-1 inoculation, ASF+Fn7-1 mice did not exhibit histological hallmarks of inflammation (Figure 3(d,e)), supporting that, in contrast with ETBF, intestinal colonization with Fn7-1 does not induce colitis. Together, these results support that the ASF gnotobiotic mouse model can be used to probe how Fn7-1, rather than broader shifts in the microbiota, specifically influences immune responses within the healthy colon, with the goal of providing insight into how Fn7-1 influences a pro-inflammatory environment.

### F. nucleatum *increases intestinal Th17 cell frequency and IL-17 expression in ASF mice*

**Figure 4.**
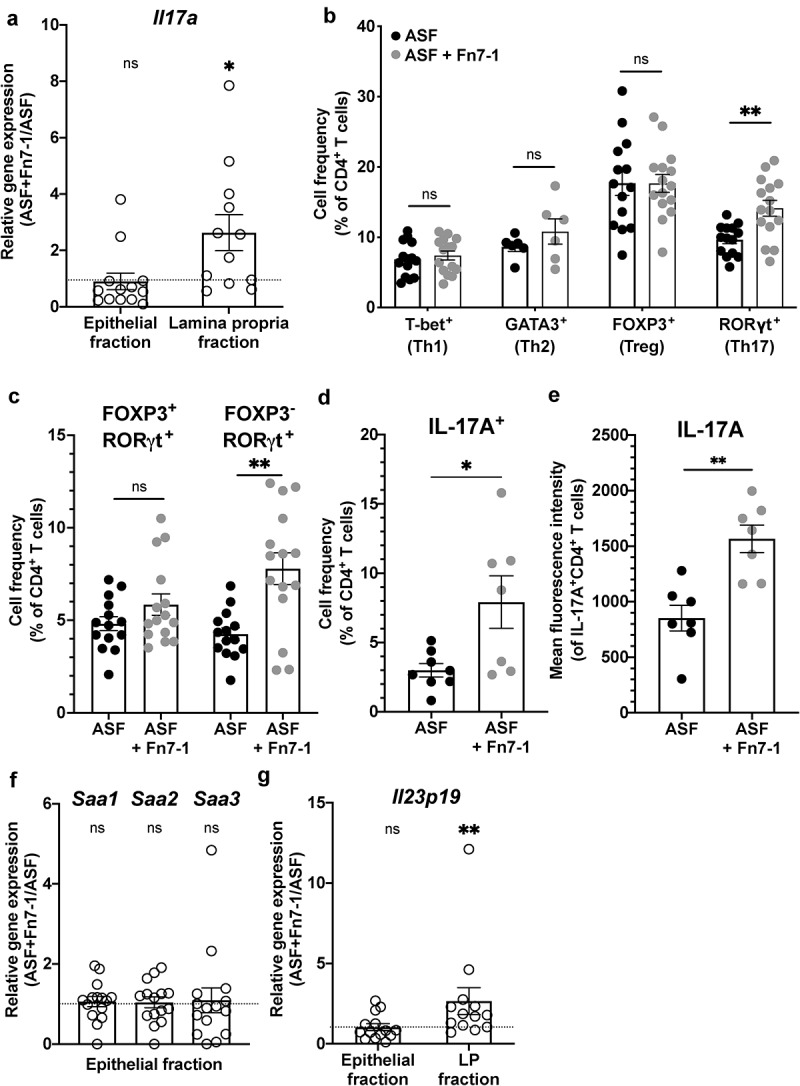
**Fn7-1 leads to a specific increase in Th17 cell frequency and IL-17A expression in the colonic lamina propria of ASF mice**. (a) *Il17a* gene expression in the colonic epithelium and LP of ASF+Fn7-1 relative to ASF mice, by RT-qPCR. (b) Frequency of T helper cell subsets in the colonic LP of ASF and ASF+Fn7-1 mice, by intracellular transcription factor staining and flow cytometry. (c) FOXP3 expression within RORγt^+^ CD4^+^ T cells distinguishes between Fn7-1 induction of Th17 and RORγt^+^ T regulatory cells in the colonic LP of ASF mice, demonstrated by the frequency of FOXP3^+^RORγt^+^ and FOXP3^−^RORγt^+^ cells within CD4^+^ T cells. (d & e) IL-17A expression, by frequency within stimulated CD4^+^ T cells (d) and mean fluorescence intensity of IL-17A within IL-17A-expressing CD4 + T cells (e) in the colonic LP of ASF and ASF+Fn7-1 mice. (f) Relative colonic epithelial expression of *Saa1, Saa2*, and *Saa3* in ASF or ASF+Fn7-1 mice, by RT-qPCR. (g) Relative colonic epithelial and LP expression of *Il23p19* in ASF or ASF+Fn7-1 mice, by RT-qPCR. For b, c, and d, CD4^+^ T cells are defined by gating on live CD45^+^CD3^+^CD4^+^ lymphocytes. All data points reflect the data from an individual mouse or sample, with error bars indicating mean ± SEM, and statistics were calculated using a Mann-Whitney test (for b, c, d, and e) and a Wilcoxon signed-rank test compared to a null hypothesis of 1 (for a, b, and g). * indicates *P* < .05, ** indicates *P* < .01; ns indicates not significant

With this ASF+Fn7-1 mouse system established, we returned to our question of how Fn7-1 might lead to development of a pro-tumorigenic environment and increased *Il17a* expression. We first measured colonic *Il17a* expression in ASF and ASF+Fn7-1 mice by quantitative reverse transcription PCR (RT-qPCR) (Figure 4a). We saw increased expression in the LP of mice colonized with Fn7-1, similar to our findings in the neonatal model. However, in this model the increase was specific to the colonic LP, without a significant increase in the epithelial fraction. To identify the cellular source of this increased *Il17a* expression, we used flow cytometry to assess the immune cells in the colonic LP from ASF or ASF+Fn7-1 mice. We first examined CD4^+^ T helper cell subsets using subset-specific transcription factor staining (Figure 4b, and Supplemental Figures S1 and S2a-c). We observed a specific increase in the frequency of RORγt^+^ cells within the CD4^+^ T cell population (Figure 4b and Supplemental Figure S2c). As we detected a slight decrease in total colonic LP CD4^+^ T cells in ASF+Fn7-1 mice, perhaps related to the shorter colons in these mice, this increased RORγt^+^ CD4^+^ T cell frequency did not lead to higher cell numbers (Supplemental Figure S2b). In previous work, we studied colonic LP cell populations from ASF mice and ASF mice colonized with *Bacteroides ovatus*, and did not observe increased colonic RORγt^+^ CD4^+^ T cell frequency in wild-type C57Bl/6J mice.^25^ Taken together, these observations support that this increased RORγt^+^ CD4^+^ T cell frequency is not induced indiscriminately by the addition of a ninth member to the ASF and represents a specific response to Fn7-1.

While we suspected the RORγt^+^ CD4^+^ T cells that changed in response to Fn7-1 were Th17 cells and could explain the increased *Il17a* expression we observed, we also examined FOXP3 expression, a marker for T regulatory cells (Tregs), within the RORγt^+^ CD4^+^ T cell population. Fn7-1 colonization led to a specific increase in FOXP3^−^ RORγt^+^ CD4^+^ T cell frequency but not FOXP3^+^ RORγt^+^ CD4^+^ T cells, consistent with this population representing Th17 cells, not RORγt^+^ Tregs (Figure 4c and Supplemental Figure S2d). To further validate that the cells affected by Fn7-1 colonization were indeed Th17 cells, we examined Il-17A expression and observed a significant increase in IL-17A^+^ frequency within the colonic LP CD4^+^ T cell population isolated from ASF+Fn7-1 mice compared to ASF (Figure 4d and Supplemental Figure S2e&f). The expression of IL-17A, as determined by mean fluorescence intensity, was also significantly increased within these cells (Figure 4e). We expanded our study of Th17 cytokine expression within the colonic LP CD4^+^ T cell populations of ASF and ASF-Fn7-1 mice to examine another IL-17 family member, IL-17F, which has previously been linked to *F. nucleatum*.^26^ We observed an increase in IL-17F^+^ CD4^+^ T cell frequency and a trend toward higher IL-17F expression, albeit in only a small number of mice (Supplemental Figure S3a-d). We could not detect IL-22, another important Th17 cytokine, by flow cytometry in these samples, and returned to RT-qPCR to assess *Il22* expression in these mice. We observed a significant increase in LP *Il22* expression in ASF+Fn7-1 mice (Supplemental Figure S3e). We also examined how Fn7-1 colonization affected other RORγt^+^ cell populations that might contribute to an IL-17-associated signature: RORγt^+^ CD8^+^ T cells (Tc17), RORγt^+^ TCRγ*δ*^+^ T cell (γ*δ*17), and RORγt^+^ innate lymphoid cells (ILC3). We observed no changes in Tc17 or ILC3 frequency or number, and, while there was increased γ*δ*17 cell frequency in many ASF+Fn7-1 mice, this observation was inconsistent and therefore not significant (Supplemental Figure S4). Together, our results suggest that, in the colonic LP, Fn7-1 specifically modulates Th17 cell frequency and the expression of the pro-inflammatory cytokines IL-17A and IL-17 F within these cells.

Two pathways have predominantly been described to explain how microbes influence Th17 cells in the murine intestinal tract: increased epithelial serum amyloid A (SAA) expression, as induced by segmented filamentous bacteria and other commensals,^27^ and induction of IL-23 signaling, as observed for enteric mouse-specific pathogens like *Helicobacter hepaticus* and *Citrobacter rodentium*.^28^ To investigate how Fn7-1 might influence these pathways, we first examined the expression of *Saa1, Saa2*, and *Saa3* in the colonic epithelium of ASF and ASF+Fn7-1 mice by RT-qPCR and observed no difference in their expression (Figure 4f). We next examined the effect of Fn7-1 colonization on *Il23p19* expression in the colonic epithelium and LP (Figure 4g) and observed increased expression in the LP of Fn7-1 colonized mice. These results suggest that Fn7-1 might be influencing Th17 responses via upstream IL-23 signaling.

### *Loss of FFAR2, a receptor of the microbial metabolites SCFA produced by* F. nucleatum in vivo, *abrogates* F. nucleatum *induction of intestinal RORγt^+^ CD4^+^ T cells*

**Figure 5.**
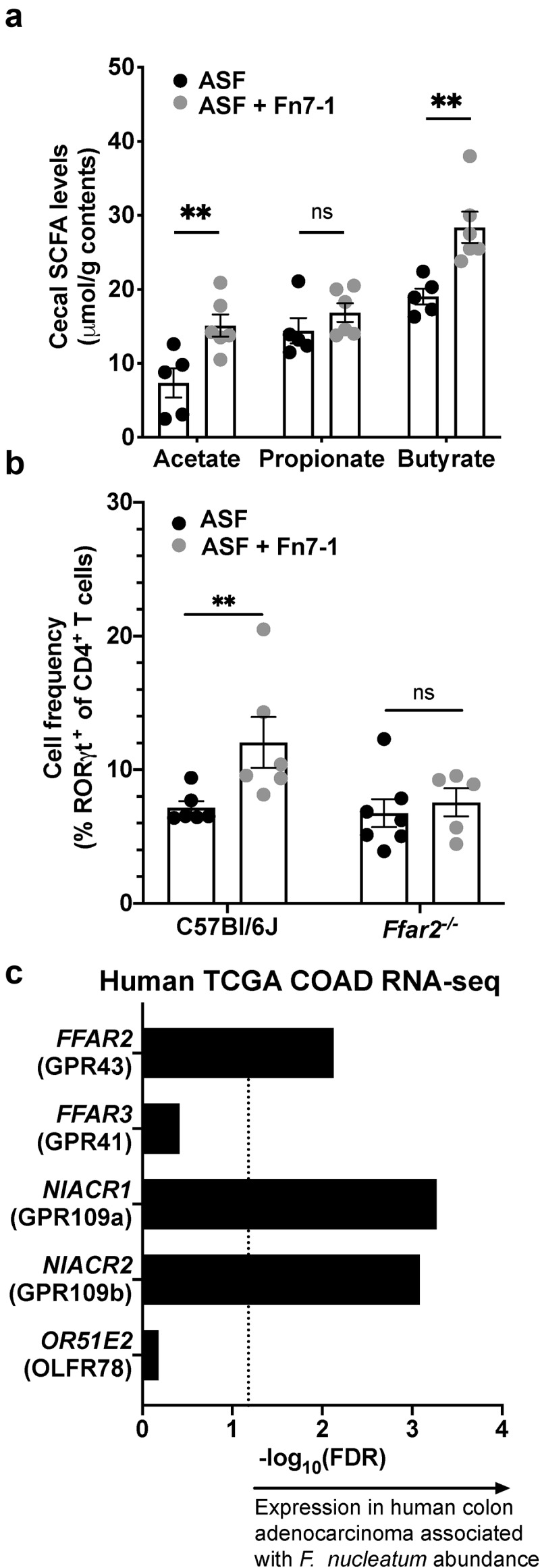
**FFAR2, a receptor for the microbial metabolites SCFA, is required for Fn7-1 induction of RORγt^+^ CD4^+^ T cells in the colonic lamina propria of ASF mice and is expressed in *F. nucleatum*-associated CRC tissues**. (a) SCFA profiles from extracted cecal contents of ASF or ASF+Fn7-1 mice and analyzed by HPLC. (b) Frequency of RORγt^+^ cells, within the CD4^+^ T cell population, in the colonic lamina propria of C57Bl/6J or *Ffar2^−/−^* mice colonized with ASF or ASF+Fn7-1, by flow cytometry. (c) Spearman’s rank correlation of SCFA receptors in human CRC tissues with intratumoral *F. nucleatum* abundance, analyzed from The Cancer Genome Atlas colon adenocarcinoma (TCGA COAD) RNA-seq data and presented as -log_10_(False Discovery Rate). For b, CD4^+^ T cells are defined by gating on live CD45^+^CD3^+^CD4^+^ lymphocytes. For a and b, all data points reflect the data from an individual mouse or sample, with error bars indicating mean ± SEM, and statistics were calculated using a Mann-Whitney test. ** indicates *P* < .01; ns indicates not significant

We next considered differences between our models and previous work that observed the *F. nucleatum*-associated proinflammatory IL-8/IL-6/TNF signature.^1,7^ A key difference is stable colonization with Fn7-1. *F. nucleatum* is known to produce large amounts of immunomodulatory short-chain fatty acids (SCFA) in response to the fermentation of amino acids and other nutritional sources.^29^ While SCFA were originally identified as inducers of Treg cells, studies have also linked SCFA to changes in other cell types, including ILC3s, Th17 cells, and neutrophils,^30–34^ leading us to posit that *F. nucleatum*-produced SCFA could play a role in the induction of Th17 cells we observed. We first asked whether Fn7-1 produced SCFA in the mouse intestinal tract, by measuring cecal SCFA levels from mice colonized with either ASF or ASF+Fn7-1 (Figure 5a). We found significant increases in both acetate and butyrate in the presence of Fn7-1, consistent with observations that these are the predominant SCFA produced by *F. nucleatum* in culture^29^ and supporting that Fn7-1 produces substantial and appreciable SCFA in the murine intestinal tract above the levels produced by the ASF members alone.

Given limitations in the genetic tractability of Fn7-1 and the redundant pathways predicted to produce SCFA in *F. nucleatum*,^35,36^ we turned to genetic mouse models to investigate whether the SCFA produced by Fn7-1 were involved in the immune responses we observed. We used mice lacking FFAR2, a SCFA receptor that senses acetate, propionate, and, to a lesser extent, butyrate, that we previously re-derived under germ-free conditions.^30^ Using the same ASF+Fn7-1 model, we asked whether Fn7-1 elicited the same induction of RORγt^+^ CD4^+^ T cells we observed in wild-type C57Bl/6J mice in the absence of FFAR2. In the colon LP of *Ffar2^−/−^* mice, RORγt^+^ CD4^+^ T cells frequency did not differ between mice harboring ASF+Fn7-1 versus ASF alone, in contrast with our observations in C57Bl/6J mice, despite similar levels of Fn7-1 colonization in both C57Bl/6J wild type and *Ffar2^−/−^* mice (Figure 5b and Supplemental Figure S5).

Our results suggest FFAR2 deficiency abrogates Fn7-1’s induction of RORγt^+^ CD4^+^ T cells, although the specific FFAR2-expressing cell type(s) mediating this phenotype have yet to be determined. We explored whether this effect might be Th17 cell-intrinsic or extrinsic by assessing *Ffar2* expression in Th17 cells isolated from colonic tissues from IL-17A-GFP reporter mice (Supplemental Figure S6). Using RT-qPCR, we found detectable *Ffar2* expression in Th17 cells, as compared to the limit of detection for *Ffar2* amplification in the absence of reverse transcriptase. We compared *Ffar2* expression in Th17 cells to colonic Treg cells, which have been shown to express *Ffar2* and respond directly to SCFA through *Ffar2,*^31^ and found that *Ffar2* expression was lower in Th17 cells than in Treg cells. As our results suggest low *Ffar2* expression in Th17 cells, it remains feasible that this phenotype could be due to either intrinsic SCFA sensing by Th17 cells or through another cellular intermediary, like certain myeloid cell populations that express high levels of *Ffar2*.^37^

The potential for FFAR2 sufficiency to mediate the immunomodulatory effects of Fn7-1 and its SCFA suggested that FFAR2 expression in *F. nucleatum*-associated tissues would be critical for the *in vivo* relevance of our observations. Previous reports investigating FFAR2 have suggested that its expression in human CRC tissues is either downregulated or similar to normal colonic tissues.^38–40^ However, these studies have not considered the tumoral microbiota in their analyses. To this end, we reexamined a data set in which we previously analyzed gene expression in human CRC tissues from The Cancer Genome Atlas Colon Adenocarcinoma data collection (TCGA-COAD)^41^ as it correlates with fusobacterial abundance.^1^ We first looked at *FFAR2* and observed significant correlation between its expression and intratumoral fusobacterial abundance (Figure 5c). Examining other genes related to SCFA sensing, we noted that the expression of *NIACR1*, which recognizes butyrate as well as nicotinic acid, and its close relative *NIACR2* were also significantly correlated with fusobacterial abundance in CRC tissues. Expression of *FFAR3* and *OR51E2*, both of which encode SCFA receptors, did not correlate with fusobacterial burden. These observations support that CRC tissues that harbor *F. nucleatum* are able to sense SCFA, suggesting that these metabolites are potentially important immunomodulators for *F. nucleatum*’s shaping of the intestinal tumor microenvironment as well.

## Discussion

*F. nucleatum* is an intriguing target for CRC diagnostics and therapeutics, particularly as epidemiological studies have found that tumoral fusobacterial load is associated with poorer patient prognosis and recurrence after treatment.^42,43^ In this work, we used murine gnotobiotic models to reveal that *F. nucleatum* colonization increases colonic *Il17a* expression, intestinal Th17 cell frequency and IL-17-family cytokine production, reminiscent of how ETBF promotes tumorigenesis and suggesting a convergent mechanism between these two CRC-associated microbes. Given its lack of obvious toxins, how *F. nucleatum* is shaping the intestinal immune environment into this pro-tumorigenic milieu remains a question of utmost importance. Our study further intimates that recognition of SCFA, metabolites produced by *F. nucleatum* in the intestinal tract, contributes to its immunomodulatory capabilities.

Th17 cells have complex roles in immune system function as they can mediate both appropriate and maladaptive responses in infectious and inflammatory disorders.^44^ Their roles in CRC models are similarly complex, exemplified by disruption of IL-17 signaling that leads to either increased or decreased tumor burden depending on the pre-clinical model.^45^ Extensive work in ETBF has revealed the pro-tumorigenic potential for this pathway in oncomicrobe-mediated intestinal tumorigenesis. In this study, we demonstrate *F. nucleatum*, another oncomicrobe, alters intestinal Th17 cell frequency and IL-17 family member cytokine expression. Although the modulation of Th17 cells we observe in response to *F. nucleatum* under otherwise homeostatic conditions is mild, it remains to be seen how this response might be exacerbated by a secondary disruption, such as chronic inflammation or antibiotic treatment. Further, while we showed *F. nucleatum* leads to increased IL-17 expression prior to tumor formation in the neonatal colonization model, the relative contribution of IL-17 signaling to the development and tuning of the tumor microenvironment in *F. nucleatum*-associated CRC remains to be determined. While beyond the scope of this work, future studies that disrupt IL-17 signaling, through genetic modification or neutralizing antibodies, in the *F. nucleatum* preclinical models we have developed herein could begin to unravel the importance of IL-17 signaling for *F. nucleatum-*positive human CRC.

We demonstrate that Fn7-1 colonization leads to increased intestinal SCFA levels and that human CRC tissues harboring *F. nucleatum* are primed to sense these immunomodulatory metabolites, with higher expression of the genes encoding the SCFA receptors *FFAR2* and *NIACR1*. We also show that *Ffar2^−/−^* mice do not exhibit increased Th17 cell frequency in the presence of Fn7-1. However, SPF *Ffar2^−/−^* mice exhibit altered fecal microbiota composition from wild-type control animals under some conditions,^39^ and, while we observed no obvious shifts in the microbiota composition of wild-type ASF mice after Fn7-1 gavage, we did not perform 16S rRNA amplicon sequencing for *Ffar2^−/−^* mice under these same conditions. Thus, it remains a formal possibility that microbiota differences in ASF *Ffar2*^−/−^ mice as compared to wild-type mice, rather than the absence of SCFA-sensing through FFAR2, prevent Fn7-1 colonization from increasing colonic Th17 cell frequency. Further, whether SCFA signaling through FFAR2 is sufficient for this phenotype remains to be seen, and, if not, it would suggest the need for a specific fusobacterial trigger in addition to this more general microbial metabolite immunomodulator. Thus, the specific mechanism underlying the role of FFAR2 in *F. nucleatum*’s induction of Th17 has not yet been clarified. Herein, we demonstrate that colonic Th17 cells do express *Ffar2*, suggesting that a Th17 cell-intrinsic mechanism is feasible, although it remains to be seen what signaling pathways downstream of FFAR2 might mediate their recruitment or differentiation. However, another intriguing hypothesis is a cell-extrinsic mechanism by which *F. nucleatum*-produced SCFA drive neutrophil chemotaxis in an FFAR2-dependent manner, as has been demonstrated for acetate.^32,33^ Neutrophils are a cellular source of intestinal IL-23,^46^ which can drive Th17 recruitment and the expression of which was increased in the colonic LP of *F. nucleatum*-colonized mice. This hypothesis is supported by two recent studies: one that described *F. nucleatum* supernatants triggered neutrophil chemotaxis in an FFAR2-dependent manner in culture^47^ and another which observed that another SCFA – propionate – mediated Th17 induction by adherent-invasive *E. coli* in a manner requiring mononuclear phagocytes.^48^ While we have previously demonstrated that *F. nucleatum* increases intratumoral myeloid cells, including tumor-associated neutrophils, *in vivo* using the daily treated *Apc^Min/+^* model,^1^ we have not yet examined the myeloid compartment in the models established and investigated herein.

SCFA, and the signaling pathways engaged by their recognition, have complicated roles in intestinal immunity and tumorigenesis. SCFA have largely been linked to the induction of immune cells, including Tregs and ILC3s, that dampen inflammation and improve intestinal barrier function. SCFA are therefore often perceived as beneficial to intestinal homeostasis, as well as more broadly on human health and metabolism.^49^ Supporting a beneficial role for SCFA in the setting of CRC is the increased intestinal tumor burden observed in *Apc^Min/+^* mice lacking either *Ffar2* or *Niacr1*.^39,40,50,51^ However, the SCFA butyrate has also been shown to stimulate colonic epithelial proliferation and to promote tumorigenesis in *Apc^Min/+^* x *Msh2^−/−^* mice.^52^ These seemingly conflicting reports underscore the importance of context (*e.g*., other mutations or environmental or microbial triggers, amount of SCFA available at a specific location and/or accessible to certain cell types) as it relates to metabolite-mediated modulation of immune responses. The gut microbiome and its metabolites have increasingly robust links not only for susceptibility to cancer but responsiveness to and toxicities experienced with chemotherapies and immunotherapies (reviewed by Sepich-Poore *et al*.^53^). As such there is a critical, unmet need for preclinical models that enable careful interrogation of oncomicrobes and their metabolites across a spectrum of questions related to cancer development and treatment. Our study, which provides tools and methods that we hope will help progress the field, suggests that, under the specific conditions of *F. nucleatum* colonization, SCFA could mediate biological effects that create a tumor-permissive immune microenvironment.

Another remaining question is what *F. nucleatum* is catabolizing in the intestinal tract that leads to SCFA production, as the pathways engaged by such nutrients may ultimately provide important insight in the metabolic state of and virulence terrain of *F. nucleatum in vivo*. Unlike intestinal *Bacteroides* spp. which metabolize fibers from the diet to SCFA, *F. nucleatum* is known to ferment amino acids, predicted to be present the intestinal tract, to SCFA. Additionally, unraveling other carbon sources and colonization factors used by Fn7-1 to establish itself in the colon may provide targets for modulation of both fecal fusobacterial burden, a marker of increased risk for CRC,^54^ and its production of SCFA through dietary interventions.

Given that CRC develops over decades and that *F. nucleatum* is specifically enriched in human CRC tissues while rarely found in healthy stool, an outstanding question in the field is when *F. nucleatum* exposure is important as it relates to shaping CRC outcomes. Therefore, a critical knowledge gap exists in differentiating the distinct contributions of *F. nucleatum* in a developing pro-tumorigenic milieu versus its roles in shaping an existing tumor microenvironment. Using neonatal inoculation of *Apc^Min/+^* mice with Fn7-1, we have developed a model in which *F. nucleatum* potentiates tumorigenesis but does not persist to colonize the resulting tumors, which can be used to dissect these distinct roles. Such tools are critical as we consider how our understanding of oncomicrobes in tumorigenesis can be transformed into either preventative or therapeutic approaches alter the treatment-refractory trajectory of *F. nucleatum*-high CRC and improve CRC outcomes.

## Materials and methods

### Bacterial strains and growth conditions

*F. nucleatum* strain Fn7-1 (also known as EAVG_0002^16^) was grown in Columbia broth or tryptic soy broth supplemented with hemin (5 μg/ml) and menadione (1 μg/ml) (sTSB) at 37°C, under anaerobic conditions using a vinyl chamber (Coy Lab Products). Fastidious anaerobe agar (FAA) supplemented with 5% defibrinated sheep blood was used for plating. For selection of *F. nucleatum*, FAA plates were supplemented with josamycin (3 μg/ml), vancomycin (4 μg/ml), and norfloxacin (1 μg/ml) (JVN).

### Animal experiments

All experiments were approved and carried out in accordance with Harvard Medical School’s Standing Committee on Animals and the National Institutes of Health guidelines for animal use and care.

- ***Neonatal inoculation of Apc^Min/+^ mice***

Specific-pathogen free (SPF) *Apc^Min/+^* and C57Bl/6J mice were sourced from Jackson Labs and bred in house in a barrier facility under a 12-hour light cycle and with *ad libitum* food and water. Pregnant SPF C57Bl/6J mice, bred to SPF *Apc^Min/+^* males, were identified at gestational day ~18 and gently orally instilled with either ~5×10^8^ CFU of Fn7-1 (in <100 μl volume) or medium control (sTSB) daily until they gave birth (generally 1–3 times). After birth, pups were orally instilled with ~1×10^8^ colony forming units (CFU) of Fn7-1 (in <20 μl volume) and sprinkled with another ~5×10^8^ CFU per litter at d14. Pups were orally instilled again, as previously at weaning (d18-28). Sham mice were instilled and sprinkled with equivalent amounts of medium control (sTSB) on the same schedule. Mice were aged until 14 weeks for tumor enumeration, or 7–9 weeks for pre-tumoral intestinal gene expression.

- ***Gnotobiotic colonization***

For gnotobiotic experiments, C57Bl/6J mice, originally sourced from Jackson Labs prior to germ-free rederivation, and germ-free *Ffar2^−/−^* mice on a C57Bl/6J background,^30^ were colonized with the altered Schaedler flora (ASF)^21^ by oral gavage. Gnotobiotic ASF mice were maintained in semirigid gnotobiotic isolators for breeding or transferred to individually ventilated isolator cages for further experimentation. For Fn7-1 colonization, male and female ASF-colonized mice were gavaged once with ~10^9^ CFU of Fn7-1 (in <200 μl volume) at approximately 8 weeks of age and aged for an additional two weeks before sacrifice. Mice colonized with Fn7-1 at below 10^3^ CFU per g feces were excluded from analysis.

### Histological analysis

For tumor enumeration, *Apc^Min/+^* mice were euthanized at 14 weeks of age, and colons and small intestines were excised. Macroscopic tumors were counted and then tissue prepared for histological assessment. For colitis assessment, mice were euthanized 2 weeks post-Fn7-1 gavage, and colons were excised, measured, prepared for histological assessment. Tissues were fixed in 4% paraformaldehyde before paraffin embedding. All histological analyses were performed by JNG on hematoxylin and eosin (H&E) stained tissue in a blinded manner, as previously described.^1^ Colitis scoring considers monocyte infiltration, hyperplasia, injury, polymorphonuclear cell infiltration, and percent involvement.

### Preparation of colonic lamina propria (LP) and epithelial samples

Colons were removed, opened longitudinally, and all contents were dissected. Fat and, for *Apc^Min/+^* colons, any macroscopic abnormalities suggestive of tumors were removed. Colons were incubated in PBS with 1 mM DTT for 10 minutes on ice. Epithelial fractions were dissociated from tissue by placing colons in 5 mM EDTA in PBS+3% fetal bovine serum (FBS) for 2 rounds of 15-minute incubations at 37°C on a rotator. LP samples were digested into single cells, when needed, by washing remaining tissue in PBS, chopping into approximately 1-mm pieces, and incubating for two subsequent rounds in digestion media (RPMI with glutamine with 10% FBS, 1% penicillin/ streptomycin, 0.5 mg/mL dispase, 1 mg/mL collagenase D, 50 mg/mL DNAse) for 30-minutes at 37°C on a rotator, as previously described.^40^ After samples were vigorously vortexed for 45 seconds, isolated single cells were passed through a 40 μm filter, washed in PBS, and resuspended as needed for further analysis.

### Gene expression

RNA was extracted from by resuspending cells or tissues in Qiazol lysis reagent (Qiagen Cat#79306) and following the manufacturer’s instructions. After RNA isolation, samples were treated with the DNA-free DNA removal kit (Invitrogen Cat#AM1906) and then used as the template for cDNA synthesis with the iScript kit (Bio-Rad Cat#1708891) per manufacturer’s instructions. Quantitative reverse transcription PCR (RT-qPCR) was performed then using the KAPA SYBR Fast kit (KAPA Cat#KK44618) and primer sets listed in Supplemental Table S1. All quantitative PCR was performed using a Stratagene Mx3005P machine (Agilent Technologies).

### F. nucleatum enumeration from stool and tissues

Fecal *F. nucleatum* abundance was determined by qPCR using the SYBR Fast kit and primers targeting *Fusobacterium* 16S and eubacterial 16S,^1^ on fecal DNA isolated by bead-beating followed by phenol-chloroform extraction. *F. nucleatum* abundance in normal and tumor tissues was determined by qPCR using the Probe Fast Low ROX kit (KAPA Cat#KK5718) with primers and probes targeting *Fusobacterium nusG*^17,18^ and mouse *ActB* on tissue DNA isolated with the Allprep DNA/RNA Mini kit (Qiagen Cat#80204). Primers and probes are listed in Supplemental Table S1.

Fecal Fn7-1 load from ASF mice was determined by weighing fecal samples, homogenizing stool in sTSB with a wide-bore pipette tip, vortexing samples for 15 seconds, and then centrifuging for 3 minutes at 2000 x *g* to pellet debris. Serial dilutions of the stool supernatant were performed and plated on JVN plates, which were placed at 37°C under anaerobic conditions. Fecal load was calculated by colony-forming units per g feces based on fecal wet weight.

### 16S rRNA sequencing of stool from ASF mice

16S rRNA amplicon sequencing was performed on stool isolated from 7 ASF mice and 9 ASF+Fn7-1 mice using protocols adapted from the Earth Microbiome Project^55^ and as previously described.^56^ From extracted fecal DNA, the 16S rRNA V4 region was amplified by PCR, with a single-indexing approach (linker primer sequence: 5ʹ-CAAGCAGAAGACGGCATACGAGAT-3ʹ and indexing barcodes as listed in Supplemental Table S2) and sequenced on a MiSeq instrument (Illumina, San Diego, CA) using 2x150bp paired-end protocol (v2 chemistry). Quality of reads was checked using FastQC (v0.11.5). Read pairs were imported to the QIIME2 environment^57^ (version 2021.2), where they were joined, denoised and checked for chimeras using the DADA2 plug-in. Operational taxonomic units (OTUs, Supplemental Table S2) were assigned by using a subset of the SILVA database^58^ (version 138.1) containing sequences from the V4 region of 16S rRNA gene. For Figure 3c and Supplemental Table S2, OTUs representing >0.5% relative reads in at least one sample were then assigned to predicted ASF member(s) based on 16S sequence. Linear discriminant analysis effect size (LEfSe) analysis was performed to identify OTU differences between ASF and ASF+Fn7-1 samples.^59^ Raw sequences were deposited in NCBI SRA databank, Bioproject PRJNA721215.

### Flow cytometry

Single-cell suspensions of LP cells were counted by hemocytometer and stained with Fixable Live/Dead Yellow (Invitrogen Cat#L34959), per manufacturer’s instructions. After washing in FACS buffer (PBS + 2% FBS, 1 mM EDTA), ~10^6^ cells were treated with FC block (10 minutes, on ice) and then stained with fluorochrome-conjugated antibodies targeting surface antigens (30 minutes, on ice). After washing, cells were permeabilized with the Biolegend FOXP3 Fix/Perm kit (Biolegend Cat#421403), per manufacturer’s instructions, and stained with intracellular antibodies for 40 minutes at room temperature, before analysis with a BD LSRII flow cytometer. All antibodies are listed in Supplemental Table S1. In parallel, cells were also stained with the respective control isotype antibodies.

For cytokine staining, cells were *ex vivo* stimulated for 4 hours in RPMI supplemented with 50 ng/mL phorbol-12-myristate 13-acetate (PMA) and 500 ng/mL ionomycin, with Brefeldin A added for the final 2 hours. After which, cells were stained as above, with the addition of Cytofix (BD Biosciences Cat#554655, RRID:AB_2869005) treatment for 10 minutes prior to the permeabilization step.

### Measurement of Ffar2 expression in colonic Th17 and Treg cells

Cells of interest were isolated from C57BL/6-*Il17a^tm1Bcgen^*/J (IL-17A reporter mice), which were generously provided by Dr. Lydia Lynch (Brigham and Women’s Hospital, Boston, MA). Colonic LP was processed into single cell suspensions, as described above, and stimulated *ex vivo* with a cocktail of PMA, ionomycin, brefeldin A and monensin (ThermoFisher Scientific, Cat#00-4975-03) at 37°C for 4 hours. Cells were subsequently stained for viability (Biolegend 7-AAD Viability Staining Solution, Cat#420404), per manufacturer’s instructions, and surface-stained with fluorescently conjugated monoclonal antibodies, as described above. Th17 cells (live, single, CD45^+^CD3^+^CD4^+^CD25^−^GFP^+^ cells) and Tregs (live, single, CD45^+^CD3^+^CD4^+^CD25^+^GFP^−^ cells) were sorted directly into RLT buffer, using a BD FACSAria IIu sorter.

For RT-qPCR, RNA was extracted using the RNEasy Mini Kit (Qiagen Cat#74106) according to manufacturer’s instructions. cDNA was prepared using SuperScript IV VILO Master Mix (ThermoFisher Scientific Cat#11756050) and amplified using TaqMan PreAmp Master Mix (ThermoFisher Scientific Cat#4384267) according to manufacturer’s instructions. The pre-amplification reaction was carried out for 14 cycles and diluted 1:10 in TE buffer. RT-qPCR reactions were prepared using the ThermoFisher Scientific TaqMan Gene Expression Assay, according to manufacturer’s instructions, with primer-probe sets listed in Supplemental Table S1.

### Cecal short-chain fatty acid (SCFA) determination

Cecal contents were collected immediately after animals were sacrificed and stored at −80°C until extraction. Thawed cecal contents were weighed, homogenized in HPLC-grade water, centrifuged to remove debris, and filtered sequentially through 0.45 and 0.22 μm filters. SCFA extraction was performed as previously described,^60^ modified from Hillman.^61^ Briefly, after spiking in valeric acid as an internal standard, volatile compounds were acidified, extracted with diethyl ether, back-extracted into sodium phosphate buffer, and re-acidified. SCFA were then analyzed using an Agilent 1200 series HPLC equipped with a Poroshell 120 SB C18 column and 0.01 M sulfuric acid as the mobile phase, and quantified as described.^31^

### Statistical analysis

Graphs and statistical analyses were generated using GraphPad Prism 9 (RRID:SCR_002798). All data points reflect data from an individual mouse or sample, with error bars indicating mean ± standard error of the mean (SEM). Unless otherwise described in figure legends, statistics were calculated using a Mann-Whitney test.

## Supplementary Material

Supplemental Material
