# Supplementary figures and images for: Fusobacterium nucleatum drives a pro-inflammatory intestinal microenvironment through metabolite receptor-dependent modulation of IL-17 expression
Source: Gut Microbes. 2021 Nov 15;13(1):1987780. doi: 10.1080/19490976.2021.1987780 (PMC8604392; doi:10.1080/19490976.2021.1987780)

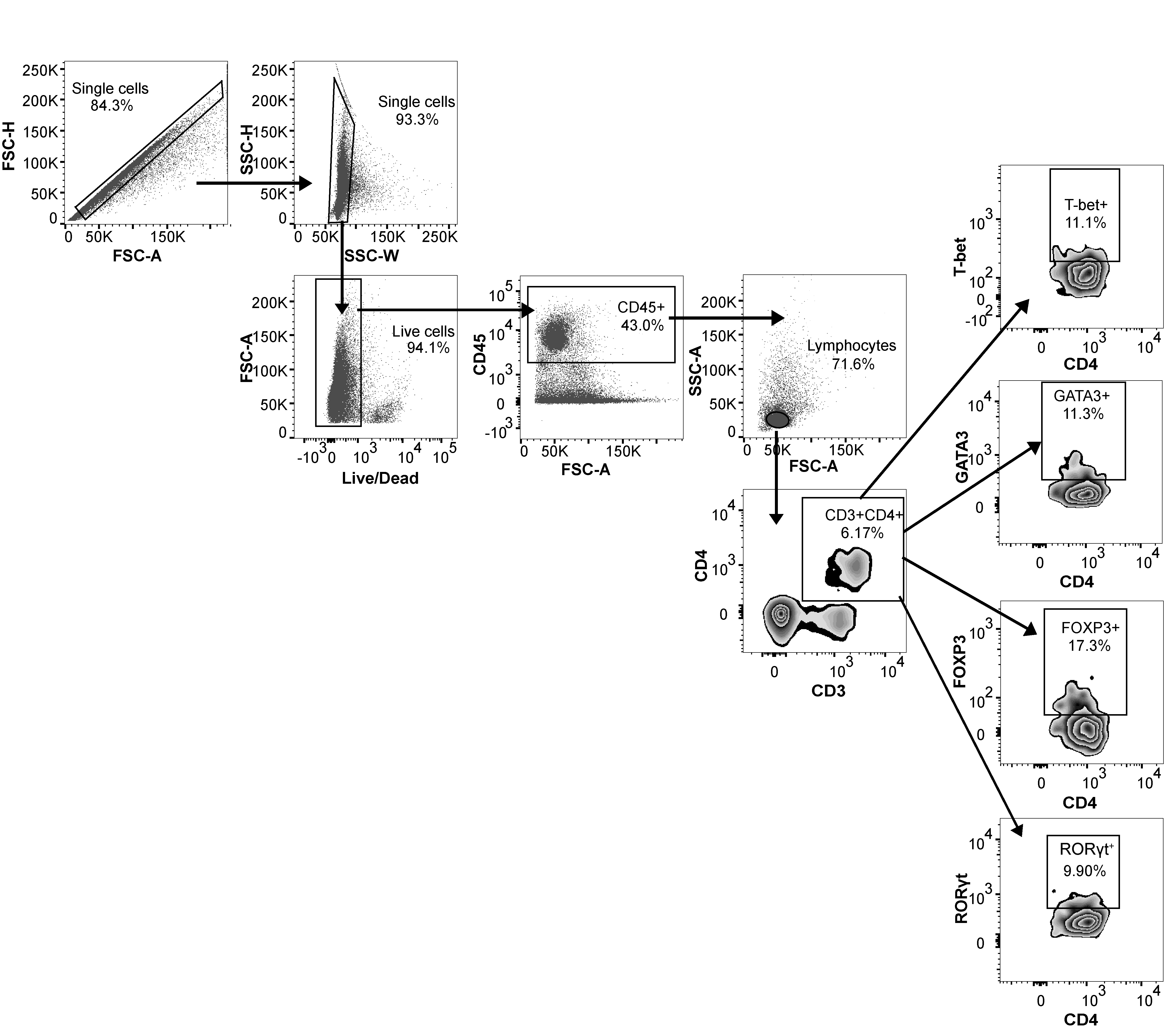

Supplement: Supplemental Material [file KGMI_A_1987780_SM4728.zip › resubTh17SuppFigS1.tif]

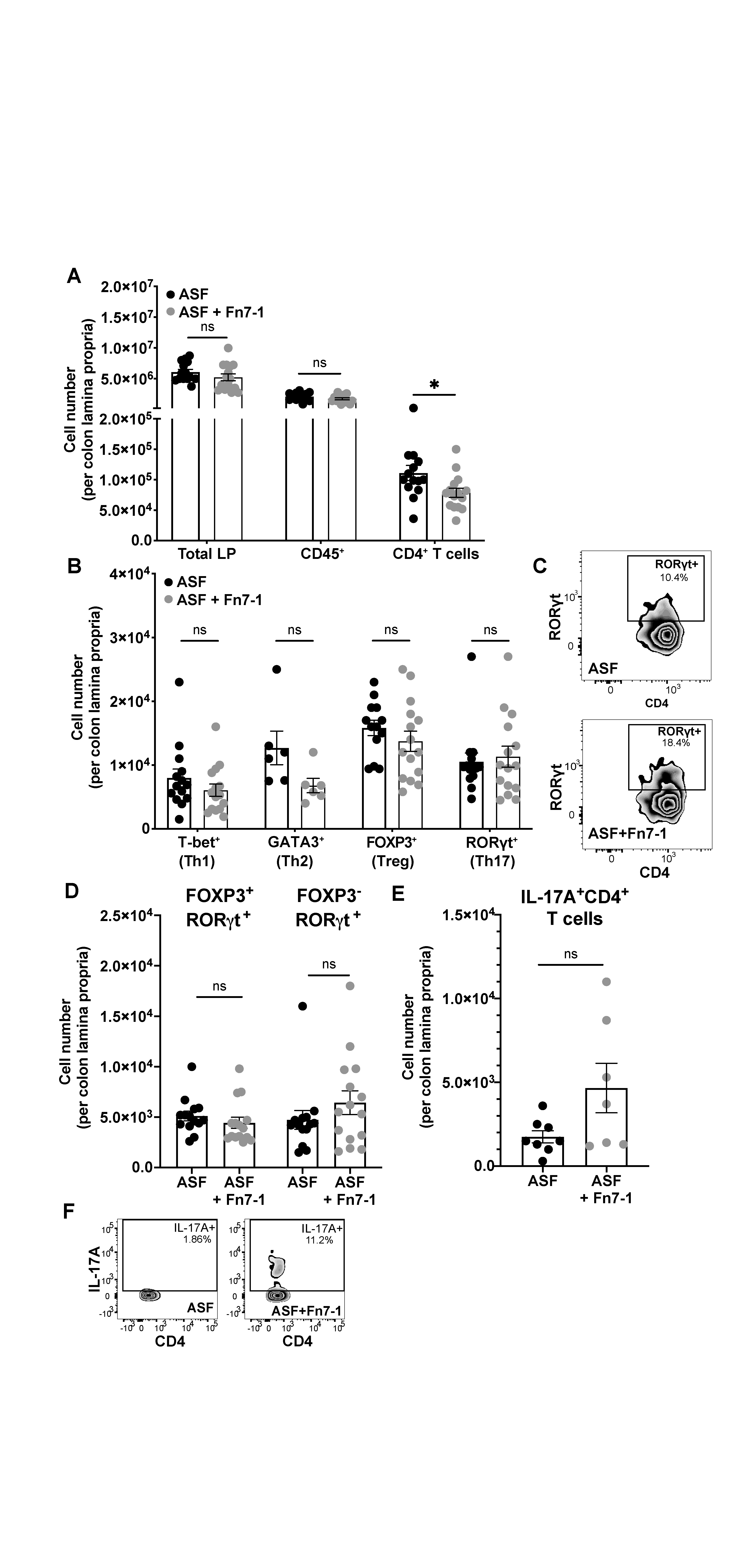

Supplement: Supplemental Material [file KGMI_A_1987780_SM4728.zip › resubTh17SuppFigS2.tif]

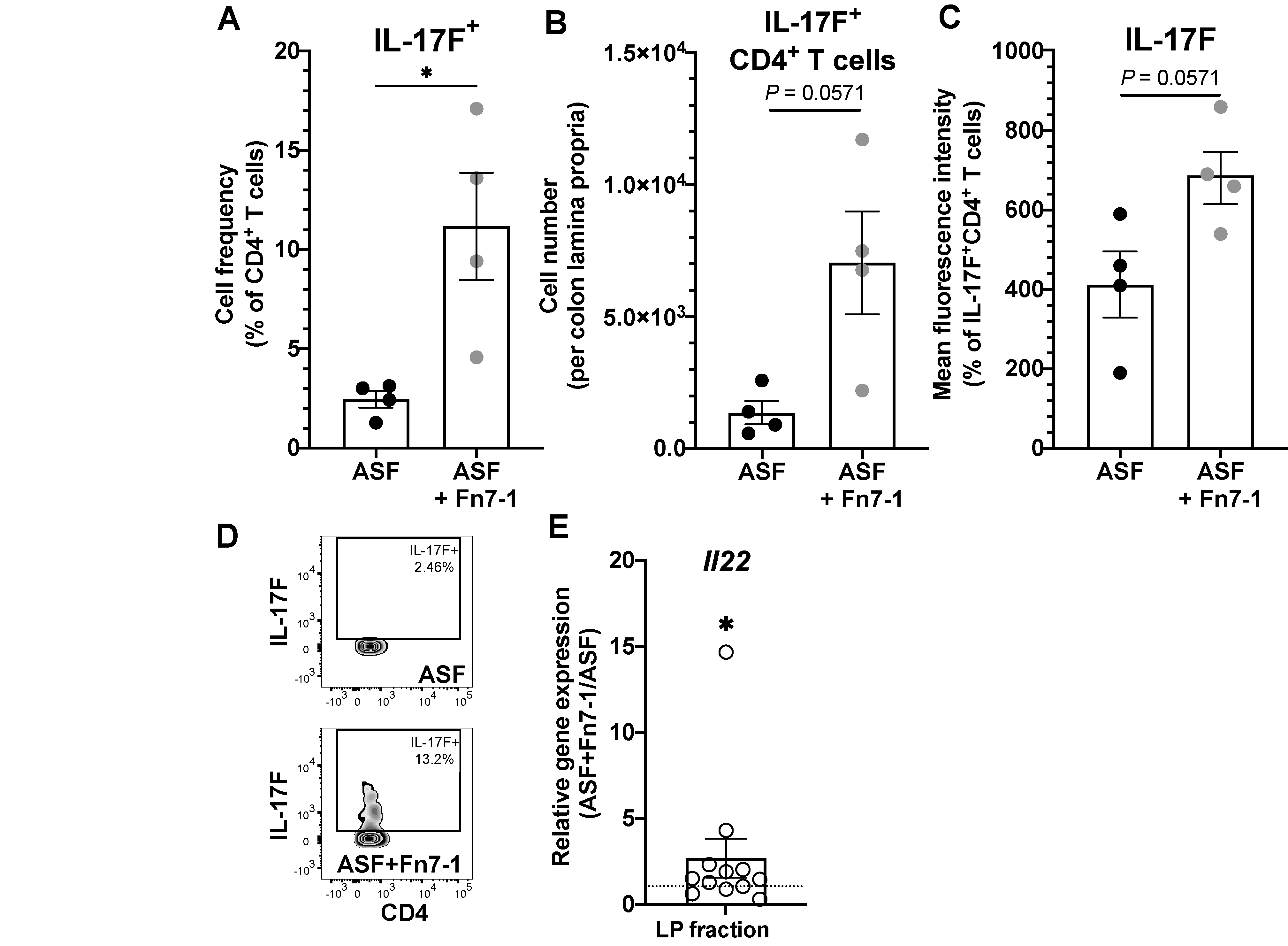

Supplement: Supplemental Material [file KGMI_A_1987780_SM4728.zip › resubTh17SuppFigS3.tif]

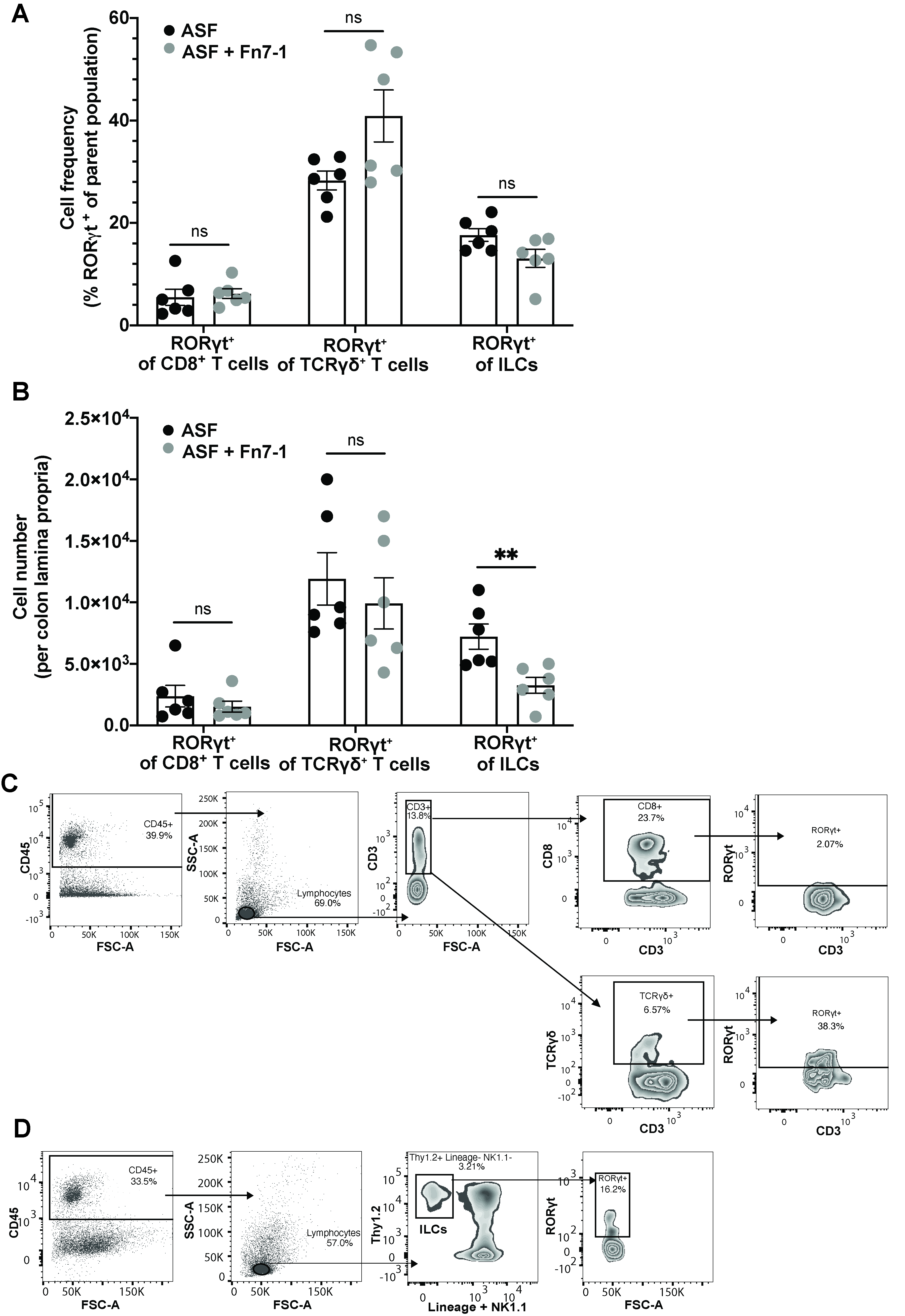

Supplement: Supplemental Material [file KGMI_A_1987780_SM4728.zip › resubTh17SuppFigS4.tif]

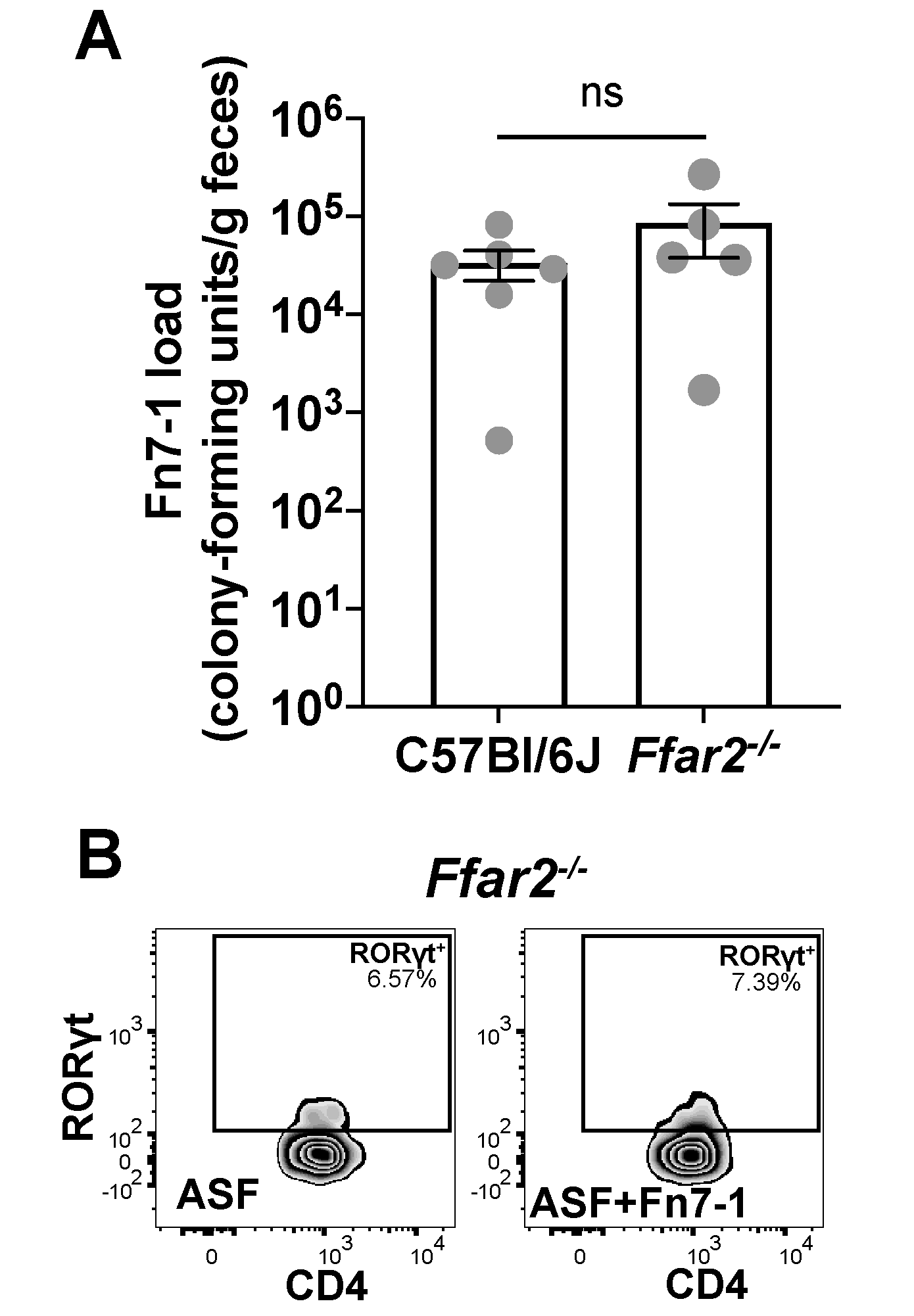

Supplement: Supplemental Material [file KGMI_A_1987780_SM4728.zip › resubTh17SuppFigS5.tif]

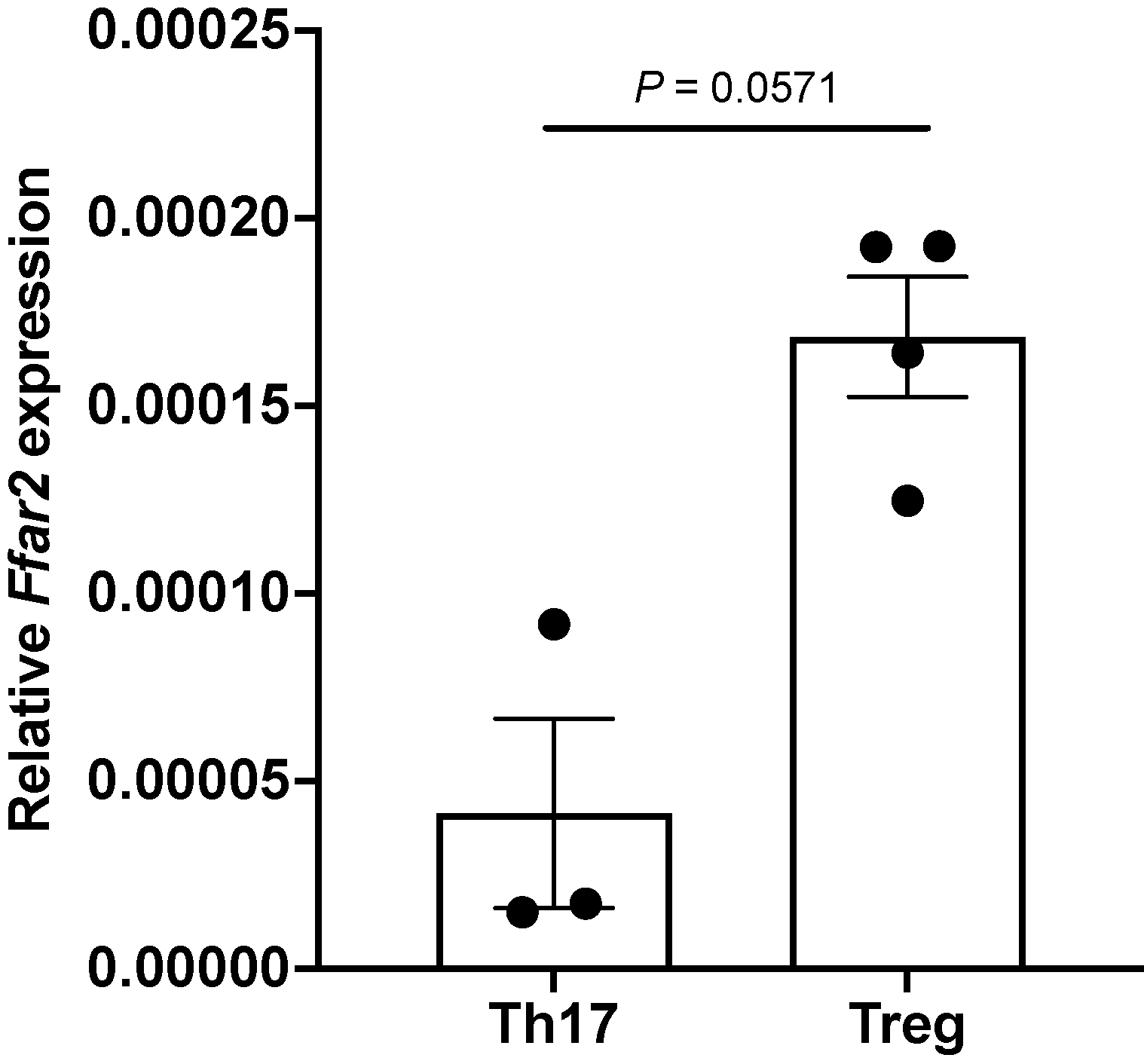

Supplement: Supplemental Material [file KGMI_A_1987780_SM4728.zip › resubTh17SuppFigS6.tif]
